# Targeting mTORC1 Activity to Improve Efficacy of Radioligand Therapy in Cancer

**DOI:** 10.3390/cancers15010017

**Published:** 2022-12-20

**Authors:** Michal Grzmil, Fabius Wiesmann, Roger Schibli, Martin Behe

**Affiliations:** 1 Center for Radiopharmaceutical Sciences, Paul Scherrer Institute, 5232 Villigen, Switzerland; 2 Department of Chemistry and Applied Biosciences, ETH Zurich, 8093 Zurich, Switzerland

**Keywords:** targeted radionuclide therapy, TRT, radioligand therapy, RLT, PRRT, mammalian target of rapamycin, mTOR, rapalogs, radiosensitization

## Abstract

**Simple Summary:**

Targeted radionuclide therapy (TRT) delivers cancer-selective radiopharmaceuticals to eradicate cancer cells while sparing healthy tissue. The recent development of combinatory treatments is a growing research field in nuclear medicine to enhance cancer cytotoxicity of TRT. Among promising combinatorial strategies, this review focuses on the rationale, efficacy, and safety of targeting the mammalian target of rapamycin complex 1 (mTORC1) to improve systemic radiation with radiolabeled ligands in cancer patients.

**Abstract:**

Radioligand therapy (RLT) represents an effective strategy to treat malignancy by cancer-selective delivery of radioactivity following systemic application. Despite recent therapeutic successes, cancer radioresistance and insufficient delivery of the radioactive ligands, as well as cytotoxicity to healthy organs, significantly impairs clinical efficacy. To improve disease management while minimizing toxicity, in recent years, the combination of RLT with molecular targeted therapies against cancer signaling networks showed encouraging outcomes. Characterization of the key deregulated oncogenic signaling pathways revealed their convergence to activate the mammalian target of rapamycin (mTOR), in which signaling plays an essential role in the regulation of cancer growth and survival. Therapeutic interference with hyperactivated mTOR pathways was extensively studied and led to the development of mTOR inhibitors for clinical applications. In this review, we outline the regulation and oncogenic role of mTOR signaling, as well as recapitulate and discuss mTOR complex 1 (mTORC1) inhibition to improve the efficacy of RLT in cancer.

## 1. Introduction to Radioligand Therapy

External beam radiation therapy (EBRT) represents the standard-of-care treatment of locally confined tumors either alone or in combination with other treatment modalities. However, patients with disseminated metastatic disease are unlikely to benefit from this approach, and systemic therapy is required for effective treatment. Targeted radionuclide therapy (TRT), such as radioligand therapy (RLT), represents another treatment strategy using ionizing radiation for spread cancer lesions by cancer-selective delivery of the radio-pharmaceuticals following systemic application [1]. RLT also allows the development of theranostic applications for personalized health care, whereby the same cancer-targeting molecule can be combined with diagnostic or therapeutic radionuclides (e.g., Galium-68 and Lutetium-177 or Copper-64 and Copper-67) with similar or even identical in vivo distribution, allowing predictive diagnosis of the therapeutic outcome [2]. The frequently overexpressed G-protein-coupled receptors (GPCRs) or other cancer-associated transmembrane proteins, which bind their ligands with high affinity [3,4], have spurred developments of RLT, including peptide receptor radionuclide therapy (PRRT) [5]. Somatostatin receptor 2 (SSTR2) belongs to the GPCR family, and it is overexpressed in the vast majority of neuroendocrine tumors. The SSTR2-targeting octreotide peptides (DOTA-TOC and DOTA-TATE) were extensively explored in clinical studies and showed very promising results for both imaging and PRRT [6,7]. Another promising target is a transmembrane glycoprotein prostate-specific membrane antigen (PSMA) that is highly expressed on prostate adenocarcinomas, contrary to normal and benign tissues. Targeting PSMA was successfully evaluated in clinical settings by using highly potent radiolabeled PSMA inhibitors such as vipivotide tetraxetan PSMA-617 [8]. Remarkably, in 2018, after successful clinical trials, the lutetium-177-labeled DOTATATE peptide (Lutathera™) was approved for the first-in-class PRRT of SSTR2-positive gastroenteropancreatic and neuroendocrine tumors [9,10], whereas in 2022, Pluvicto™ (Lu-177 vipivotide tetraxetan, formerly 177Lu-PSMA-617) was registered for the RLT of PSMA-positive metastatic castration-resistant prostate cancers [11].

Another emerging target that belongs to the GPCR family is the cholecystokinin B receptor (CCKBR, also known as CCK2R). It is highly expressed in human cancers, particularly in medullary thyroid cancer (MTC), as well as in small-cell lung cancer, astrocytoma, and stromal ovarian cancer [12], and its activation and involvement in cancer cell proliferation were shown in colorectal and pancreatic cancers [13,14]. The pioneer clinical studies with CCKBR-specific radiolabeled minigastrin analog [^177^Lu]Lu-PP-F11N demonstrated tumor-specific accumulation with low adverse reactions in MTC patients [15,16]. These studies suggest further development of the minigastrin analog for theranostics application in patients with CCKBR-positive tumors [17,18]. Likewise, a high level of gastrin-releasing peptide receptor (GRPR) was found in various malignancies including prostate and breast cancers, as well as in gliomas. Additional development of radiolabeled bombesin derivatives, which bind GRPR with high affinity, revealed great potential for the assessment of GRPR expression and PRRT [19,20,21].

Nevertheless, despite recent therapeutic successes of the RLTs, many patients still do not respond to the treatment. Insufficient delivery of the radioactive compounds into cancer lesions and radioresistance of malignant tumors, as well as cytotoxicity to healthy organs, significantly limit the efficacy in the clinic [22,23]. Thus, observed restricted cure rates reveal the need for further development of treatment protocols. Notably, on top of the curative potential of the standard-of-care EBRT, the administration of simultaneous targeted therapies against the oncogenic signaling pathways that support growth, vascularization, and survival has been shown to enhance locoregional control and survival rates in different cancer patients [24]. In contrast, most of the RLTs are given as a monotherapy. Thus, the exploration of synergistic combination therapies is a highly promising and growing area of RLT optimization to potentiate cancer-selective cytotoxicity of systemic radiation [25,26,27]. This multimodality approach can be beneficial by either radiosensitizing, increasing tumor uptake, or modulating the microenvironment and immune system to improve disease management with minimal increase in the toxicity to healthy organs. Among promising combinatorial strategies, our review focuses on targeting mTORC1 signaling pathways to improve RLT in cancer.

## 2. Overview of Regulation and Signaling of mTORC1 in Cancer

mTOR is a serine/threonine kinase, discovered as a mechanistic (or mammalian) target of rapamycin, and it functions in two distinct complexes. These complexes are distinguished by the interaction partners, subcellular localization, and substrate specificity, as well as by the sensitivity to rapamycin [28,29]. mTOR complex 1 (mTORC1) comprises the regulatory associated protein of mTOR (RAPTOR), which acts as a scaffold and interacts with inhibitory protein proline-rich AKT substrate 40 kDa (PRAS40). mTOR complex 2 (mTORC2) contains scaffold protein, the rapamycin-insensitive companion of mTOR (RICTOR), which recruits regulatory protein MAPK-interacting protein 1 (mSIN1) and the protein associated with RICTOR 1 and 2 (PROTOR1/2). Both mTORC1 and mTORC2 contain lethal sec-13 protein 8 (mLST8/GβL), which stabilizes the kinase domain, and can interact with inhibitory protein DEP-domain containing mTOR-interacting protein (DEPTOR).

**Figure 1:**
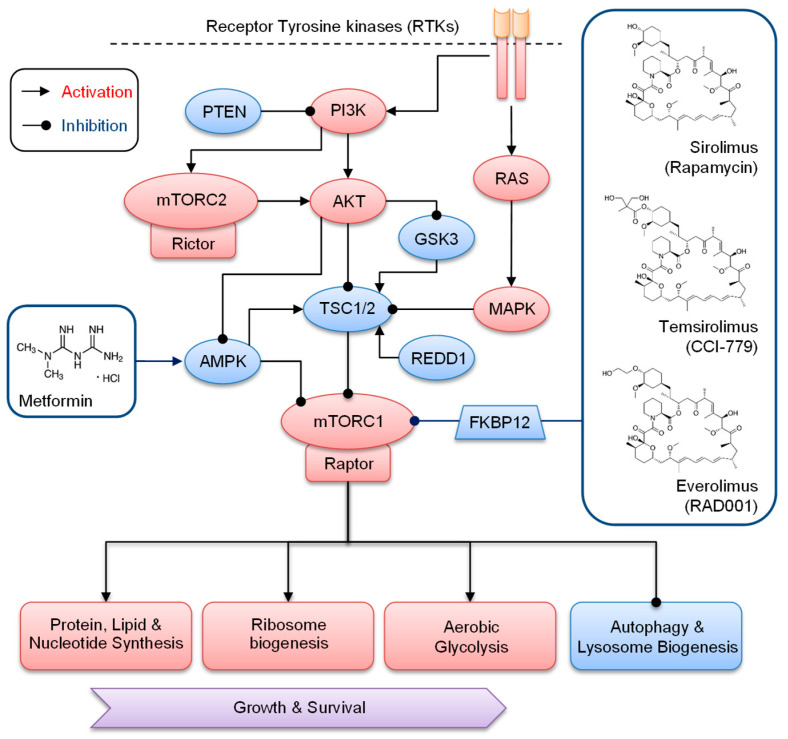
Regulation and targeting mTORC1 by rapalogs and metformin in cancer. Associated with raptor or rictor, mTOR kinase functions in two distinct complexes: mTORC1 and mTORC1, respectively. In cancers, hyperactivated RTKs together with downstream PI3K/AKT, RAS, and MAPK pathways activate mTORC1 via phosphorylation and inactivation of TSC1/2. In proliferating cells, PI3K-mediated phosphorylation of mTORC2 increases AKT activity, which in turn inhibits TSC1/2 regulators, AMPK, and GSK3. During energy stress, such as hypoxia, REDD1 inhibits mTORC1 activity via TSC1/2. Frequent mutations of PTEN, which antagonizes PI3K, keep AKT in the active state. Hyperactivated mTORC1 promotes cancer growth and survival by the stimulation of protein, lipid, nucleotide, and ribosome synthesis, aerobic glycolysis, and inhibition of autophagy and lysosome biogenesis. Sirolimus (rapamycin), temsirolimus (CCI-779), and everolimus (RAD001) inhibit mTORC1 by binding to FKBP12, which in turn interacts with mTORC1 and deters its autophosphorylation and activation. Metformin inhibits mTORC1 by activating AMPK, which activates TSC1/2 and inactivates mTORC1.

In human cancers, overexpressed or hyperactivated via oncogenic mutation receptor tyrosine kinases (RTKs), phosphatidylinositol-3 kinase (PI3K), and v-akt murine thymoma viral oncogene (AKT), as well as small GTPase (RAS) and mitogen-activated protein kinase (MAPK) signaling pathways, converge to activate mTORC1 (Figure 1). This regulation occurs via phosphorylation and inactivation of the major mTORC1 inhibitory complex, the tuberous sclerosis complex (TSC), composed of TSC1 (hamartin) and TSC2 (tuberin) [30]. TSC1/2 acts as a GTPase-activating protein for Ras-homolog enriched in the brain (Rheb), and its inactivation enhances the level of active Rheb-GTP, a key molecule required for mTORC1 activation [31,32]. In addition, phosphorylated mTORC2 via PI3K increases AKT activity by the phosphorylation of the AKT hydrophobic motif [33] and enhances activation of the mTORC1. Activated AKT can further sustain mTORC1 activity via phosphorylation and inactivation of the AMP-activated protein kinase (AMPK) and glycogen synthase kinase 3 (GSK3), which can inhibit mTORC1 directly and/or via TSC1/2 complex [34,35,36]. On the other hand, a tumor suppressor phosphatase and tensin homolog (PTEN) can inhibit mTORC1. PTEN antagonizes PI3K activity via the regulation of a second messenger in the plasma membrane, which recruits phosphoinositide-dependent kinase 1 (PDK1) required for AKT activation [37]. Nevertheless, in human cancers, frequent PTEN mutations keep AKT in an active state [38]. In addition, in response to energy stress, such as hypoxia, regulation in DNA damage and development 1 (REDD1) inhibits mTOR function via activating the TSC1/2 complex [39]. Similarly, DNA damage leads to mTORC1 inhibition via induction of the transcription factor p53, which increases expression of the mTORC1 negative regulators including TSC2, AMPK, and PTEN [40].

The mTORC1 promotes carcinogenesis by supporting growth, proliferation, and survival. Hyperactivated mTORC1 positively regulates protein synthesis by phosphorylation of eIF4E-binding proteins (4E-BPs), which in hyperphosphorylated states release translation initiation factor E (eIF4E) for cap-dependent translation [41]. Furthermore, mTORC1-mediated phosphorylation of scaffold protein eIF4G and S6 kinase (S6K), which regulates ribosomal protein S6 and eIF4B factor, as well as mTORC1-regulated transcription of RNA polymerase I and III, further supports ribosome biogenesis and protein synthesis [42,43]. In addition, signaling of the S6K enhances the level of the translation elongation factor eEF2 and leads to proteasomal degradation of the translation inhibitor programmed cell death 4 (PDCD4), which blocks RNA helicase activity of the eIF4A, required for unwinding highly structured 5′UTRs of cancer-promoting mRNAs [44,45]. In the highly proliferating cancer cells, mTORC1 together with S6K promotes lipid and nucleotide synthesis via sterol responsive element binding proteins (SREBPs), which act as transcription factors for fatty acid and cholesterol biosynthesis genes [46,47]. Likewise, mTORC1 hyperactivation increases nucleotide biosynthesis via activating transcription factor 4 (ATF4)-dependent expression of methylenetetrahydrofolate dehydrogenase 2 (MTHFD2), required for purine synthesis [48]. However, mTORC1-activated S6K phosphorylates and activates carbamoyl-phosphate synthetase 2, aspartate transcarbamylase, and dihydroorotase (CAD), which are essential for the pyrimidine synthesis [49,50].

During cancer progression, reduced oxygen supply to the expanding tumors leads to the hypoxia condition, whereby mTORC1 further supports tumor growth by changing oxidative glucose metabolism to prevalent in carcinomas aerobic glycolysis [51]. Signaling of mTORC1 increases expression of hypoxia-inducible factor 1 alpha (HIF1α), which is a transcription factor that regulates gene expression of glycolytic enzymes and glucose transporters involved in the glycolytic flux control [52]. In addition, HIF1α increases the transcription of vascular endothelial growth factors (VEGFs), which facilitate tumor vascularization and cancer progression [53]. The proliferation and growth are also supported by mTORC1 signaling, which negatively regulates autophagy and biogenesis of lysosomes, induced by nutrient deficiency and starvation. The autophagy lysosomal pathways are involved in the degradation of cytotoxic, dysfunctional, or non-essential intracellular components, whereby the obtained biomolecules can be recycled in stress conditions [54]. mTORC1 phosphorylates and inhibits key components of the autophagy induction such as unc-51-like kinase 1 (ULK1) and the mammalian ATG13 protein, as well as disrupts autophagosome maturation by the phosphorylation of UV radiation resistance-associated (UVRAG). Furthermore, mTORC1 phosphorylates transcription factor EB (TFEB), as well as the related transcription factor E3 (TFE3), and inhibits their nuclear translocation, required for the expression of the lysosomal biogenesis genes [55,56].

## 3. Inhibitors of mTOR for Cancer Therapy

The first generation of clinical mTORC1 inhibitors were derived from rapamycin, which was isolated from Streptomyces hygroscopicus, and named after its place of origin, Easter Island, called Rapa Nui in the native language [57]. Initially, rapamycin (sirolimus) was explored as an antifungal agent, and after the discovery of its immunosuppressive and antitumor properties, it was eventually developed in the clinic. The mTORC1 inhibitor sirolimus was approved for the prevention of organ transplant rejections and lymphangioleiomyomatosis (LAM), a rare progressive lung disease. Rapamycin, and successively developed rapamycin analogs (rapalogs) including RAD001 (everolimus) and CCI-779 (temsirolimus), are allosteric inhibitors, which deplete mTORC1 activity by binding to the immunophilin 12-kDa FK506-binding protein (FKBP12), which then interacts with the FKBP12-rapamycin binding (FRB) domain of mTOR and deters its autophosphorylation and activation [58,59]. In rapamycin-insensitive mTORC2, the large scaffold protein RICTOR hinders the binding of FKBP12-rapamycin to mTOR. Nevertheless, the long-term rapamycin or rapalog treatment leads to the binding of FKBP12-rapamycin to the newly synthesized mTOR kinases, preventing mTORC2 formation and resulting in the gradual decline of mTORC2 [60].

In the clinic, rapalogs showed cytostatic rather than cytotoxic effects, and their therapeutic use was limited to certain types of cancer [61]. Both everolimus and another mTORC1 inhibitor temsirolimus were registered for advanced kidney cancer therapy. Furthermore, everolimus was approved for the treatment of advanced and unresectable cancers, including subependymal giant cell astrocytoma (SEGA), HER2-negative breast cancer, and progressive or metastatic pancreatic, gastrointestinal, and lung neuroendocrine tumors (NET) [62]. The AMPK-activating drugs such as metformin, phenformin, or A-769662 can also indirectly inhibit mTORC1 activity [63]. Markedly, metformin is registered as an antidiabetic drug and shows promising anticancer efficacy and safety profiles in various human cancers [64]. It interferes with the mitochondrial respiration pathways, leading to an increase in the cellular AMP level, which subsequently results in AMPK activation. Notably, rapalog-mediated inhibition of mTORC1/S6K1 signaling increases the level of insulin receptor substrate-1 (IRS1) and facilitates hyperactivation of the survival-promoting PI3K/AKT pathway [65]. Thus, to diminish PI3K signaling, developed dual inhibitors such as PI-103, NVP-BEZ235, GSK2126458, XL765, or SF1126 target both mTORC1/2 and PI3K [66]. These inhibitors displayed a more potent apoptotic activity as compared with the rapalogs and showed promising benefits in clinical trials for the treatment of hematological and solid cancers [67].

In addition to mTORC1 inhibition, the second generation of ATP-competitive inhibitors such as PP242, Torin1/2, WYE-132, Ku-0063794, OSI027, or AZD8055 depletes mTORC1/2 activity in cancer cells [67,68]. Although they showed superiority to the rapalogs in inhibition of mTOR downstream pathways or/and therapeutic efficacy, tissue cytotoxicity might limit their applications. Thus, further clinical studies are currently validating their tolerability at effective doses. More recently, developed bivalent mTOR inhibitor RapaLink combines features of both rapalogs and ATP-competitive inhibitors by simultaneous binding to the FRB (via FKBP12) and the kinase domain of mTOR [69]. This study also demonstrated high sensitivity to RapaLink-1 of both rapamycin and AZD8055-resistant tumors in animal models, yet the efficacy and safety warrant further investigation in cancer patients.

## 4. Targeting mTORC1 for Improved RLT

Allosteric TORC1 inhibitors exert an anticancer activity and were shown to sensitize cancer cells to the conventional EBRT in various cancer models, including Ras-transformed cells [70], advanced renal carcinoma [71], breast cancer [72], PTEN-null prostate cancer cells [73] and, more recently, in all five tested neuroendocrine neoplasm (NEN) cell lines [74]. The radiosensitizing effect of mTORC1 inhibition can be partially explained by the autophagy induction and amplification of the autophagic cell death pathways in irradiated tumors [73]. In certain cancer cells, rapalogs induced radiosensitivity by the cell cycle arrest at the G2/M phase, in which cancer cells are more sensitive to the ionizing radiation via accumulation of the unrepaired DNA damages shortly before cellular division [72,75].

**Figure 2:**
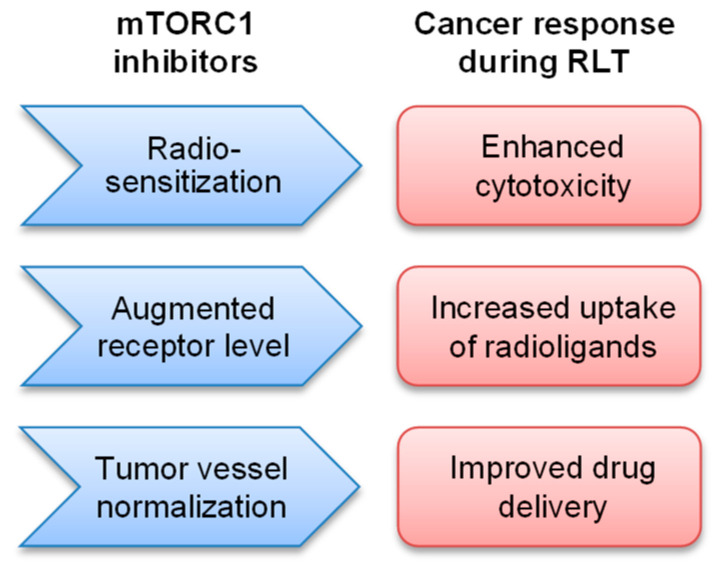
Potential of mTORC1 inhibitors to improve the efficacy of cancer RLT. Inhibition of mTORC1 sensitizes cancer cells and the microenvironment to ionizing radiation and enhances cytotoxicity. In addition, mTORC1 inhibitors can increase the level of the target receptors, as well as normalize tumor blood vessels, leading to increased cellular uptake and drug delivery.

Furthermore, in murine models, mTORC1 inhibition decreased tumor vascular density [76] and radiosensitized vascular endothelium [77], indicating that additionally to cancer cells, mTORC1 inhibition can also radiosensitize the cancer microenvironment. Notably, in the rapamycin-treated breast cancer mouse model, mTORC1 inhibition significantly enhanced the efficacy of chemotherapy with paclitaxel due to increased drug penetration throughout the normalized tumor vessels [78]. It suggests that mTORC1 inhibition can increase the perfusion and delivery of the radiolabeled ligands into the tumors, yet this point awaits further studies. To improve the therapeutic ratio of RLT, the mTORC1 inhibitor rapamycin was combined with radiolabeled GRPR antagonist [^177^Lu]Lu-RM2 in nude mice bearing prostate cancer PC-3 tumors [79]. In the latter, the combinatory treatment led to significantly longer survival as compared with the monotherapies without treatment-related toxicity, suggesting further development of rapalogs as radiosensitizers for RLT (Table 1). Interestingly, a trend for increased radioligand uptake was observed in the rapamycin-treated tumors, suggesting that in addition to the radiosensitizing activity, rapalogs can enhance the delivered radioactive dose to the cancer cells (Figure 2).

Similarly, in the previous in vitro study, rapamycin markedly upregulated PSMA expression and cellular uptake of radioligand [^177^Lu]Lu-PSMA-617 in androgen-dependent prostate cancer LNCaP cells [80]. These results are in line with the results described more recently, whereby kinase library screen identified AKT/mTORC1 inhibitors, which increased uptake of radiolabeled minigastrin [^177^Lu]Lu-PP-F11N in human A431/CCKBR cells [81]. In the latter, mTORC1 inhibition by everolimus increased the level of CCKBR in the cancer cells and enhanced tumor-specific uptake of radiolabeled minigastrin [^177^Lu]Lu-PP-F11N in the A431/CCKBR xenograft nude mouse model. Importantly, the radioligand uptake in healthy organs including the stomach, which expresses CCKBR, and the kidney were not influenced by the everolimus treatment. Notably, in the same study, indirect mTORC1 inhibitor metformin significantly increased the uptake of [^177^Lu]Lu-PP-F11N in A431/CCKBR cells. However, in vivo validation showed only a marginal increase in tumor uptake, which was not statistically significant. These findings suggest that metformin is less potent in vivo, and further research is needed to provide evidence of the effectiveness of metformin treatment in combination with RLT. Nevertheless, mTORC1 inhibition by everolimus has the potential to substantially improve the response of CCKBR-positive cancers to RLT. Indeed, a subsequent study in the same animal model demonstrated significantly improved therapeutic efficacy and extended survival of the everolimus and [^177^Lu]Lu-PP-F11N-treated mice as compared with the monotherapies without significant adverse effects [82]. On the other hand, a study in Lewis rats with rat pancreatic CA20948 tumors was less encouraging, whereby simultaneous everolimus and [^177^Lu]Lu-DOTATATE treatment did not produce benefit over the monotherapy, and the metastases were found both in the combination and everolimus-treated groups, suggesting that everolimus could promote malignancy [83]. Notably, further investigations in the same animal model showed that metastases were not dose-dependent or related to the duration of the everolimus treatment [84]. The latter suggests that this distinctive effect can be explained by the everolimus-mediated changes to the immune system that could be unique in this preclinical non-human tumor model. A subsequent preclinical toxicity study of the combined therapy with everolimus and [^177^Lu]Lu-DOTATATE in Levis rats did not indicate increased renal or hematological toxicity as compared with the monotherapies, prompting further characterization of the efficacy of this combination [85].

As a registered drug, everolimus is often used in clinics for the treatment of certain cancers. Early evaluation of the safety profile of everolimus in 24 patients with gastroenteropancreatic neuroendocrine tumors (GEP-NETs) showed that it was not influenced by the previous treatment with [^177^Lu]Lu-Octreotate [86]. Partial response and stable diseases were achieved in 16.7 and 62.5% of patients, respectively, whereas 12.5% of patients had progressive disease. Consistently, in the more recent study, pretreatment with everolimus and/or receptor tyrosine kinase (RTK) inhibitor sunitinib had no significant effects on the subacute hematotoxicity of [^177^Lu]Lu-DOTATATE in 41 patients with NETs [87]. Notably, a recent case study of a patient with grade II, well-differentiated rectal NET demonstrated everolimus-induced SSTR overexpression, which enabled the second course of RLT [88]. Furthermore, a phase I clinical study was initiated to define dose-limiting toxicity of neuroendocrine tumor therapy with lutetium-177-octreotate and everolimus (NETTLE) in 16 patients with advanced unresectable progressive well-differentiated GEP-NETs [89]. The combination treatment was administered with manageable and reversible toxicity at the maximum tolerated dose of 7.5 mg everolimus daily for 24 weeks, whereas at 10 mg daily dose, patients required dose reduction or complete cessation. Overall, tumor responses were observed at each dose level of everolimus, with 7 of 16 patients achieving an overall response of 44%, and no patient showed progression during the 6-month treatment period. Notably, four out of five pancreatic NET patients reached partial response (80%). Similarly, in a recent study in 11 patients with grade 1–2 NET of different origins, the combination of everolimus at a dose of 10 mg daily with [^177^Lu]Lu-DOTATATE was terminated due to toxicity in three patients and progression in another three patients [90]. One patient achieved partial response, and nine had stable disease. In summary, the clinical studies together with previous animal studies support further development of RLT in combination with everolimus. Nevertheless, the combinatory treatment warrants a larger clinical trial at a lower and optimized dose of everolimus.

**Table 1:** Combination of RLTs and mTORC1 inhibitors in animal and clinical studies.

Inhibitor	RLT	Cancer	Results	Ref.
Preclinical in vivo studies				
Rapamycin	[^177^Lu]Lu-RM2	Human prostate cancer PC-3 xenograft mouse model	Significantly longer survival as compared with the monotherapies without treatment-related toxicity	[79]
Everolimus	[^177^Lu]Lu-DOTA-PP-F11N	Human epidermoid carcinoma A431/CCKBR xenograft mouse model	Everolimus significantly enhanced tumor-specific uptake of radiolabeled minigastrin without adverse effects	[81]
Metformin	[^177^Lu]Lu-DOTA-PP-F11N	Human epidermoid carcinoma A431/CCKBR xenograft mouse model	Metformin did not increase tumor uptake of radiolabeled minigastrin	[81]
Everolimus	[^177^Lu]Lu-DOTA-PP-F11N	Human epidermoid carcinoma A431/CCKBR xenograft mouse model	Increased tumor growth inhibition and extended survival as compared with the monotherapies without adverse effects	[82]
Everolimus	[^177^Lu]Lu-DOTA-TATE	Rat pancreatic cancer model with CA20948 tumors	Lack of therapeutic benefit. Metastases were found in combination and everolimus-treated groups	[83]
Everolimus	[^177^Lu]Lu-DOTA-TATE	Rat pancreatic cancer model with CA20948 tumors	Metastases were not dose-dependent or related to the duration of everolimus treatment	[84]
Everolimus	[^177^Lu]Lu-DOTA-TATE	Lewis rats: non-tumor bearing	Everolimus did not increase renal and hematological toxicity of the RLT	[85]
Clinical studies				
Everolimus	[^177^Lu]Lu-DOTA-TATE	Retrospective study; 24 patients with well- and moderately differentiated GEP-NETs (grades 1, 2)	The safety profile of everolimus was not influenced by the previous RLT. Partial responses (16.7%), stable disease (62.5%), and progressive disease (12.5%).	[86]
Everolimus	[^177^Lu]Lu-DOTA-TATE	Single-center retrospective study; 41 patients with NETs (80% with primary GEP-NETs)	No statistically significant differences in severe subacute hematotoxicity of RLT were seen in the everolimus pretreated group as compared with the untreated group	[87]
Everolimus	[^177^Lu]Lu-DOTA-TATE	Case study of a patient with grade II, well-differentiated rectal NET	Everolimus-induced SSTR overexpression, making the patient eligible for a second course of RLT	[88]
Everolimus	[^177^Lu]Lu-DOTA-TATE	Clinical phase I; 16 patients with advanced unresectable progressive well-differentiated GEP-NETs	The maximum tolerated dose of everolimus was 7.5 mg daily. Overall response (44%) and no progression during 6-month treatment. Partial response (80%) in pancreatic NET patients	[89]
Everolimus	[^177^Lu]Lu-DOTA-TATE	Clinical phase I/II; 11 patients with grade 1–2 NET of different origin	Everolimus at a dose of 10 mg daily with RLT was terminated due to toxicity and progression. One patient achieved partial response, and nine had stable disease. One patient developed disease progression.	[90]

## 5. Conclusions and Outlook

In human cancers, hyperactivated mTORC1 signaling pathways support carcinogenesis and survival at multiple levels, as well as drive radiotherapy resistance. Thus, targeting mTORC1 represents a promising strategy for the development of combinatory treatments to improve the therapeutic efficacy of RLT. To efficiently deplete mTORC1 activity during RLT, the genetic background of the cancer patients should be taken into consideration before treatment. This will allow the exclusion of the potential non-responders with not only a low level of the targeted receptor but also with mTORC1 inhibitor-insensitive cancers. The rapalog resistance can be determined by assessing the overexpression or activation of the mTORC1 substrates, including S6K1 and 4EBP1 or regulated proteins, such as cyclin D1 and HIF-1α [91,92,93]. Research in this direction will help to develop effective personalized cancer treatments. Furthermore, combinations of RLT with the second and third generation of mTOR inhibitors, which deplete mTOR activity in both complexes, or with PI3K/mTOR dual inhibitors, warrant further safety and efficacy study in animal models, as well as in the clinic. In addition to radiosensitizing potential, mTORC1 inhibitors were shown to enhance radioligand tumor uptake, presumably by increasing the level of the target receptor on the cancer cells or by influencing tumor vascularization and improving drug perfusion and accumulation of the therapeutic compound. Nevertheless, the underlying molecular mechanisms are not fully understood and require further research. Validation of these findings for other receptors and radioligands in various cancer models, as well as complementary in vivo optimization, should further explore the therapeutic potential and feasibility of the combinatory treatments for clinical applications.
